# Camouflaging in Autistic Adults is Modulated by Autistic and Neurotypical Characteristics of Interaction Partners

**DOI:** 10.1007/s10803-024-06481-5

**Published:** 2024-07-23

**Authors:** Ren Funawatari, Motofumi Sumiya, Toshiki Iwabuchi, Tomoko Nishimura, Hidetsugu Komeda, Atsushi Senju

**Affiliations:** 1 United Graduate School of Child Development, Osaka University, Kanazawa University, Hamamatsu University School of Medicine, Chiba University, University of Fukui, Osaka, Japan; 2 Research Center for Child Mental Development, Hamamatsu University School of Medicine, Hamamatsu, Japan; 3 College of Education, Psychology and Human Studies, Aoyama Gakuin University, Tokyo, Japan

**Keywords:** Camouflaging, Masking, Social strategy, Double empathy theory, Social interaction

## Abstract

Many autistic people reportedly engage in camouflaging to navigate everyday social interactions; however, the function of this behavior remains largely unknown. We hypothesized that autistic people camouflage more toward neurotypical others than toward autistic others, employing it as a strategy to “fit in” within the neurotypical-majority community. This study aimed to empirically investigate this hypothesis for the first time. Autistic and neurotypical participants took part in a web-based survey. Data from 48 autistic and 137 neurotypical participants were analyzed. Camouflaging toward autistic and neurotypical others was separately measured using the modified Camouflaging Autistic Traits Questionnaire (CAT-Q). For each CAT-Q item, a sentence describing a hypothetical interaction partner with autistic or neurotypical characteristics was added, creating respective sentence conditions. The interaction effect of the participants’ characteristics and sentence conditions was analyzed using a multilevel regression analysis, accounting for differing individual baselines. The analysis revealed an interaction effect between participants’ characteristics and sentence conditions. The autistic group showed significantly more camouflaging in the autistic sentence condition than in the neurotypical sentence condition. Conversely, the neurotypical group did not differ significantly in camouflaging levels in the sentence conditions. Contrary to our hypothesis, autistic people demonstrated more camouflaging toward autistic others than toward neurotypical others. This finding questions the assumption that autistic people camouflage to assimilate into a neurotypical-majority society. Instead, it could be conceptualized as a more general social strategy used by autistic people aiming to improve their relationships with others.

Autistic people often have difficulties in developing or maintaining interpersonal relationships in daily life, such as in school or at work. Autism, which is defined as autism spectrum disorder in clinical psychiatry, is a neurodevelopmental condition characterized by challenges in social communication and interaction, difficulties with interpersonal relationships, and engagement in restricted or repetitive behaviors, interests, and activities (American Psychiatric Association, 2022).

To navigate these day-to-day social situations, some autistic people strategically exhibit and maintain “neurotypical” behaviors in front of others, either by hiding their autistic characteristics or by compensating for social difficulties through alternative cognitive and behavioral strategies (Hull et al., 2017; Sumiya et al., 2018). This behavior is referred to as camouflaging (Cook et al., 2021). Camouflaging plays an important role in the quality of life of autistic people both positively and negatively. For some, camouflaging becomes a successful adaptive strategy that helps them feel more integrated into the society without losing their true selves (Loo et al., 2023). However, for others, camouflaging becomes burdensome, making it difficult and tiring to continuously engage in efforts to assimilate into their social surroundings (Livingston et al., 2019). Some studies have reported that camouflaging can lead to clinical symptoms such as depression and anxiety (Hull et al., 2021). Understanding the social function of camouflaging is imperative considering the reported positive and negative effects on the well-being of autistic people.

Among the many unanswered questions about camouflaging, one implicit (and untested) assumption is that camouflaging is directed *from* autistic people *toward* neurotypical others. Two major reasons support this assumption. First, camouflaging is assumed to serve as a bridge in communication between autistic and neurotypical individuals, enabling autistic individuals to navigate social situations. The definition of camouflaging involves displaying “neurotypical” behaviors, aiming to adapt to the predominantly neurotypical social world (Cook et al., 2021), supporting such an assumed function. Therefore, camouflaging is assumed to be primarily directed from autistic people toward neurotypical others rather than toward autistic others. Second, this assumption aligns with the proposed double empathy theory, which argues that communication difficulties between autistic and neurotypical individuals result from a “disjuncture in reciprocity between two differently disposed social actors” (Milton, 2012, p. 884) rather than being attributed solely to autistic people. This theory suggests that the interplay between autistic and neurotypical characteristics of interaction partners affects their perceived difficulty in social communication. This “disjuncture” between autistic and neurotypical individuals, or the relative lack thereof between autistic people, has been observed in multiple empirical quantitative studies. Gernsbacher et al. (2017) reported that autistic people found it more difficult to interact with neurotypical others than with autistic others, mirroring the difficulties of neurotypical individuals in interacting with autistic others, supporting this theory at the individual level. Crompton et al. (2020) further supported this theory at the group level, demonstrating that groups composed solely of autistic and neurotypical individuals showed similar levels of information transfer accuracy. In contrast, accuracy decreased in groups composed of autistic and neurotypical individuals. These bodies of theoretical and empirical research support the assumption that camouflaging is primarily directed from autistic individuals toward neurotypical others.

However, this assumption has never been empirically tested. The most extensively tested and widely used measure to quantitatively assess camouflaging is the camouflaging autistic traits questionnaire (CAT-Q; Hull et al., 2019), which has been adapted for several languages (Bureau et al., 2023; Dell’Osso et al., 2022; Remnélius & Bölte, 2023; van der Putten et al., 2023), including Japanese (Hongo et al., 2022). The CAT-Q uses items asking respondents to imagine themselves in various social situations. For example, one item states, “When I interact with someone, I deliberately copy their body language or facial expressions.” However, the targets of these imaginary interactions––in this case, “someone”––are not specified in any way. A previous study suggested that when the characteristics of the target are unspecified, both autistic and neurotypical individuals tend to imagine neurotypical others as targets in questionnaires (Gernsbacher et al., 2017). Therefore, previous studies using CAT-Q might have implicitly examined how autistic and neurotypical individuals camouflage toward neurotypical others, rather than with autistic others.

In this study, we aimed to examine how the combination of autistic and neurotypical characteristics in interaction partners influences camouflaging. We hypothesized that autistic and neurotypical individuals would engage in less camouflaging when interacting with those with similar characteristics and more when interacting with those with dissimilar characteristics. Thus, autistic individuals camouflage more toward neurotypical others, whereas neurotypical individuals camouflage more toward autistic others. To investigate this hypothesis, we attempted to measure camouflaging by autistic or neurotypical participants toward autistic and neurotypical others separately using a modified CAT-Q that specifies the interaction partner as autistic or neurotypical by adding a sentence describing the respective characteristics. A series of multilevel regression analyses were conducted using the obtained dataset to examine the interaction between participant characteristics (autistic or neurotypical) and the characteristics of the interaction partners specified in the items to test the hypothesis.

## Methods

In this study, we used the Japanese version of the social responsiveness scale (SRS-2; Kamio et al., 2017) to create short sentences describing hypothetical interaction targets with autistic or neurotypical characteristics (e.g., “There are people who take things too literally”). These sentences were placed before the CAT-Q items. Most previous studies investigating the effects of neurodevelopmental characteristics between interaction partners (e.g. Crompton et al., 2020; Gernsbacher et al., 2017) have used a declarative approach by verbally indicating that the interaction target is autistic or neurotypical (e.g., “I like being around autistic people”) or having participants identify themselves in face-to-face experimental settings. However, most of these previous studies have been conducted in Western cultures. Our study is one of the first to examine this effect in a Japanese population; thus, cultural differences must be considered. Given the comparatively higher stigma levels (Someki et al., 2018) and lower acceptance levels regarding autism (Keating et al., 2023), Japanese autistic people may be less inclined to disclose their autistic identity, possibly leading to limited or no experience interacting with others who explicitly identify as autistic. Considering these cultural differences, we preferred the present characteristic-based methodology employed in previous studies within the Japanese culture (Komeda et al., 2013, 2015, 2019).

The modified CAT-Q items, combined with sentences created from the SRS-2 indicating the autistic or neurotypical characteristics of the interaction partner, were administered to autistic and neurotypical participants. We examined the hypothesis that autistic individuals camouflage more toward neurotypical others, and less toward autistic others, by conducting a series of multilevel regression analyses to examine the interaction between participants’ characteristics (autistic or neurotypical) and the characteristics of the interaction partners specified in the items.

### Participants

Fifty-seven autistic participants (AS group) were recruited from organizations supporting individuals with neurodevelopmental disorders, including support centers, employment transition support offices, disability organizations, and peer groups, alongside referrals from autistic participants. Additionally, 146 neurotypical participants (NT group) were recruited through Cross Marketing, Inc., an academic survey company in Tokyo, Japan. Neurotypical participants were group-matched for age, sex, education, and prefecture of residence (Table 1).

**Table 1:** Characteristics of participants

Characteristics	AS group (*n* = 48)	NT group (*n* = 137)	*p*
Sex (%)			.511^a^
Female	29 (60%)	90 (66%)	
Male	19 (40%)	47 (34%)	
Age (*SD*)	36.5 (10.4)	41.0 (16.8)	.177^b^
Education (%)			.100^c^
Graduate School	4 (8.3%)	5 (3.6%)	
University	28 (58%)	72 (53%)	
Junior College	1 (2.1%)	14 (10%)	
Vocational School	8 (17%)	9 (6.6%)	
College of Technology	0 (0%)	2 (1.5%)	
High School	7 (15%)	30 (22%)	
Junior High School	0 (0%)	4 (2.9%)	
Other	0 (0%)	1 (0.7%)	

^a^ Pearson’s chi-square test. ^b^ Wilcoxon rank sum test. ^c^ Fisher’s exact test

Each participant was asked if they had consulted a psychiatrist or another professional for help with neurodevelopmental disorders. Those who consulted were asked to self-report whether they had received a clinical diagnosis of autism. Participants in the AS group were excluded if they had not received a diagnosis (*n* = 7) or were unsure about their diagnosis (*n* = 2) to ensure diagnostic clarity within the participant groups. Participants in the NT group were also excluded if they reported having received a diagnosis of autism (*n* = 2) or had consulted professionals about neurodevelopmental disorders (*n* = 7). Finally, 48 autistic participants and 137 neurotypical participants met the inclusion criteria, and their data were included in the analyses.

The study obtained ethical approval from the Aoyama Gakuin University Research Ethics Committee (reference AO 20–31) and adhered to the principles of the Declaration of Helsinki. Before completing the questionnaires, participants received an information sheet and were given the option to complete a consent form. Only those who completed the consent form were granted access to the survey.

All self-reported measures for autistic and neurotypical participants were collected via a web-based survey facilitated by Cross Marketing, Inc. (Tokyo, Japan) between July and October 2021.

### Measures

#### Modified Camouflaging Autistic Traits Questionnaire (CAT-Q: Hull et al., 2019)

The CAT-Q is a widely used measure to assess camouflaging in autistic people, comprising three subscales: masking (hiding autistic characteristics, e.g. “I monitor my body language or facial expressions so that I appear relaxed”), compensation (actively trying to compensate for social difficulties through alternative, often more deliberate strategies, e.g. “When interacting with someone, I consciously copy their body language or facial expressions”), and assimilation (feeling the need to change oneself or social situations to better fit the environment, e.g. “In social situations, I feel like I’m “performing” rather than being myself”).

**Fig. 1:**
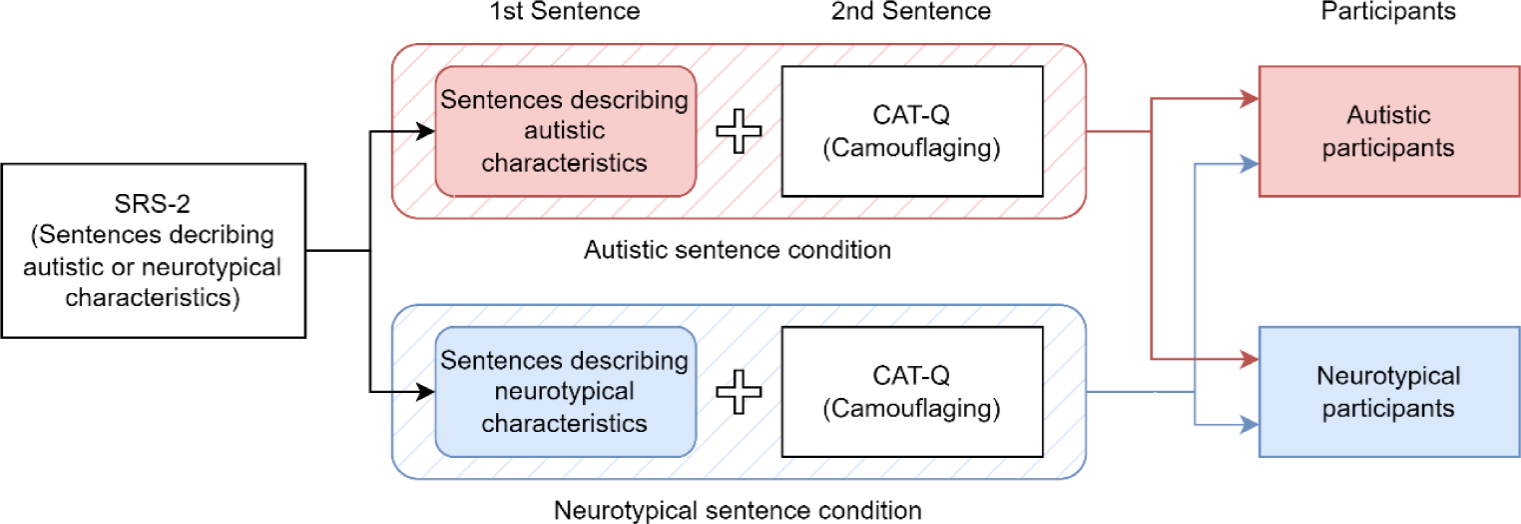
Development and administration of modified Camouflaging Autistic Traits Questionnaire (CAT-Q). Fifteen sentences describing autistic characteristics and 15 sentences describing neurotypical characteristics were created from the 30 SRS-2 items. These 30 SRS-2 items were semi-randomly selected to include six items from each of the five subscales. All CAT-Q items were used, with five items semi-randomly repeated to include five items from each of the three subscales and match 30 SRS-2 items. The combination of SRS-2 and CAT-Q items was random. The SRS-2 and CAT-Q sentences were modified to ensure a natural connection between them

As discussed in the Introduction, the CAT-Q lacks a description of the characteristics of the hypothetical interaction partner, or the hypothetical interaction partner is not mentioned in the item sentences. To examine how participants camouflage toward others with autistic or neurotypical characteristics, sentences describing the characteristics of the hypothetical interaction partner were created using the Japanese version of the SRS-2 (Kamio et al., 2017), a screening test for autism spectrum disorder. Sentences specifying the characteristics of the hypothetical interaction partner using SRS-2 (Sentence #1) and the sentences from the Japanese version of CAT-Q (Hongo et al., 2022; Sentence #2) were combined to create modified CAT-Q items measuring target-specific camouflaging (e.g., “There are people take conversation too literally [Sentence #1]. *When I have a conversation with these people*, I feel like I’m “performing” rather than being myself [Sentence #2]).” Thus, the modified CAT-Q specified the autistic and neurotypical characteristics of a hypothetical interaction partner (Fig. 1). Following the original CAT-Q procedure, responses were rated on a 7-point Likert scale ranging from “strongly disagree” to “strongly agree,” with higher scores indicating higher levels of self-reported camouflaging. The modified CAT-Q was expanded from the original 25 to 30 items to include equal amounts of each CAT-Q and SRS-2 subscale and equal autistic and neurotypical sentence conditions. An additional five items were created by randomly repeating the CAT-Q items as needed.

To create descriptions of hypothetical interaction partners, 30 SRS-2 items were used to create sentences describing autistic or neurotypical characteristics, resulting in 15 sentences for each condition. If the original SRS-2 item described the designated trait (e.g., for the autistic sentence condition: “I sometimes make the mistake of walking between two people who are trying to talk to one another” [describing an autistic characteristic]), the original item was used to create a sentence describing hypothetical interaction partners with the designated trait (e.g., “There are people who sometimes make the mistake of walking between two people who are trying to talk to one another” [describing an autistic trait]). Conversely, if the original SRS-2 item described the opposite of the designated trait (e.g., for the neurotypical sentence condition: “I concentrate too much on parts of things rather than seeing the whole picture” [describing an autistic trait]), the item was reversed to create a sentence describing hypothetical interaction partners with the designated trait (e.g., “There are people who concentrate on seeing the whole picture rather than parts of things” [describing a neurotypical characteristic]). The CAT-Q was then modified to be the succeeding sentence, adding a small section to connect the two sentences (“When I have a conversation with these people, I feel like I’m “performing” rather than being myself”). Appendix 1 provides the full list of the modified CAT-Q items.

The modified CAT-Q items, which include autistic and neurotypical sentence conditions, were administered to the AS and NT groups, creating four dyadic conditions based on the characteristics of the participants and hypothetical interaction partners: Neurotypical participants in neurotypical sentence condition, neurotypical participants in autistic sentence condition, autistic participants in neurotypical sentence condition, and autistic participants in autistic sentence condition.

To assess the reliability of this modified measure, Cronbach’s alpha was calculated. Internal consistency was good (α = 0.89). It was similar to that of the original British study (α = 0.94; Hull et al., 2019), and the study using the Japanese-translated version (α = 0.87 for autistic people; α = 0.89 for neurotypicals, Hongo et al., 2022). Internal consistencies measured separately for the AS sentence condition (α = 0.76) and the NT sentence condition (α = 0.77) were also acceptable.

### Analysis

In this study, each participant had two different scores: the modified CAT-Q score for the autistic sentence condition and the modified CAT-Q score for the neurotypical sentence condition. These scores were nested within individuals, meaning that a difference in baseline scores could confound the analysis. Therefore, multilevel regressions were used to examine the interaction effect of the participants’ characteristics and sentence conditions on camouflaging to eliminate the effect of potential differences between individual baselines. To examine whether the presence or absence of an interaction effect was consistent after controlling for participants’ sex and age, two models on the modified CAT-Q were considered (Table 2): one with no covariates (Model 1), and the other with age and sex as covariates (Model 2).

**Table 2:** Multilevel regressions of Modified CAT-Q scores and participants’ characteristics/sentence condition effects

	Model 1 (no covariates)			Model 2 (with covariates)		
		95% CI			95% CI	
Variables	β	*LL*	*UL*	β	*LL*	*UL*
Participants’ Characteristics ^a^	0.64***	0.33	0.95	0.69***	0.38	1.00
Sentence Condition ^a^	0.11	− 0.00	0.23	0.11	− 0.00	0.23
Participants’ Characteristics × Sentence Condition	0.25*	0.02	0.47	0.25*	0.02	0.48
Sex ^b^				− 0.13	− 0.41	0.15
Age				0.14	0.00	0.27

*Note* *N* = 185 (AS group: *n* = 48; NT group, *n* = 137). Multilevel regression was used to account for the different baseline scores of each participant, with the participant as a random intercept. Model 1 was a raw model with only the main and interaction effects of participants’ characteristics and sentence conditions. Model 2 was controlled for sex and age in addition to the variables included in Model 1

CI = confidence interval; *LL* = lower limit; *UL* = upper limit

^a^ NT = 0, AS = 1. ^b^ Female = 0, male = 1

*p < .05, **p < .01, ***p < .001

We used the models above to examine the association between the total score on the modified CAT-Q and participants’ characteristics. However, solely examining these models did not exclude the possibility that certain individual modified CAT-Q items, together with sentence conditions created from SRS-2, had exceedingly strong effects and skewed the analysis. We performed an additional analysis to evaluate how individually modified CAT-Q items were associated with participants’ characteristics to ensure that this skew did not affect the analysis. Ordinal logistic regression was performed for each modified CAT-Q item by comparing scores between the AS and NT groups (Appendix 2). The false discovery rate method was used for multiple comparison corrections (Glickman et al., 2014).

All analyses were performed using R (R Core Team, 2021) with the following additional packages: “tidyverse” (Wickham et al., 2019) for overall data processing and visualization, “lmerTest” (Kuznetsova et al., 2017) for multilevel regression, “ordinal” (Christensen, 2022) for ordinal logistic regression, “ggdist” (Kay, n.d.) for visualizing modified CAT-Q score distribution, and “ltm” (Rizopoulos, 2007) for calculating internal consistency for the modified measure.

## Results

### Multilevel Regressions of the Association Between Modified CAT-Q Score and Participants’ Characteristics/Sentence Condition

Two multilevel regression models were used to investigate the interaction effect of participants’ characteristics and sentence conditions on camouflaging (Table 2), with no covariates (Model 1), and sex and age as covariates (Model 2).

**Fig. 2:**
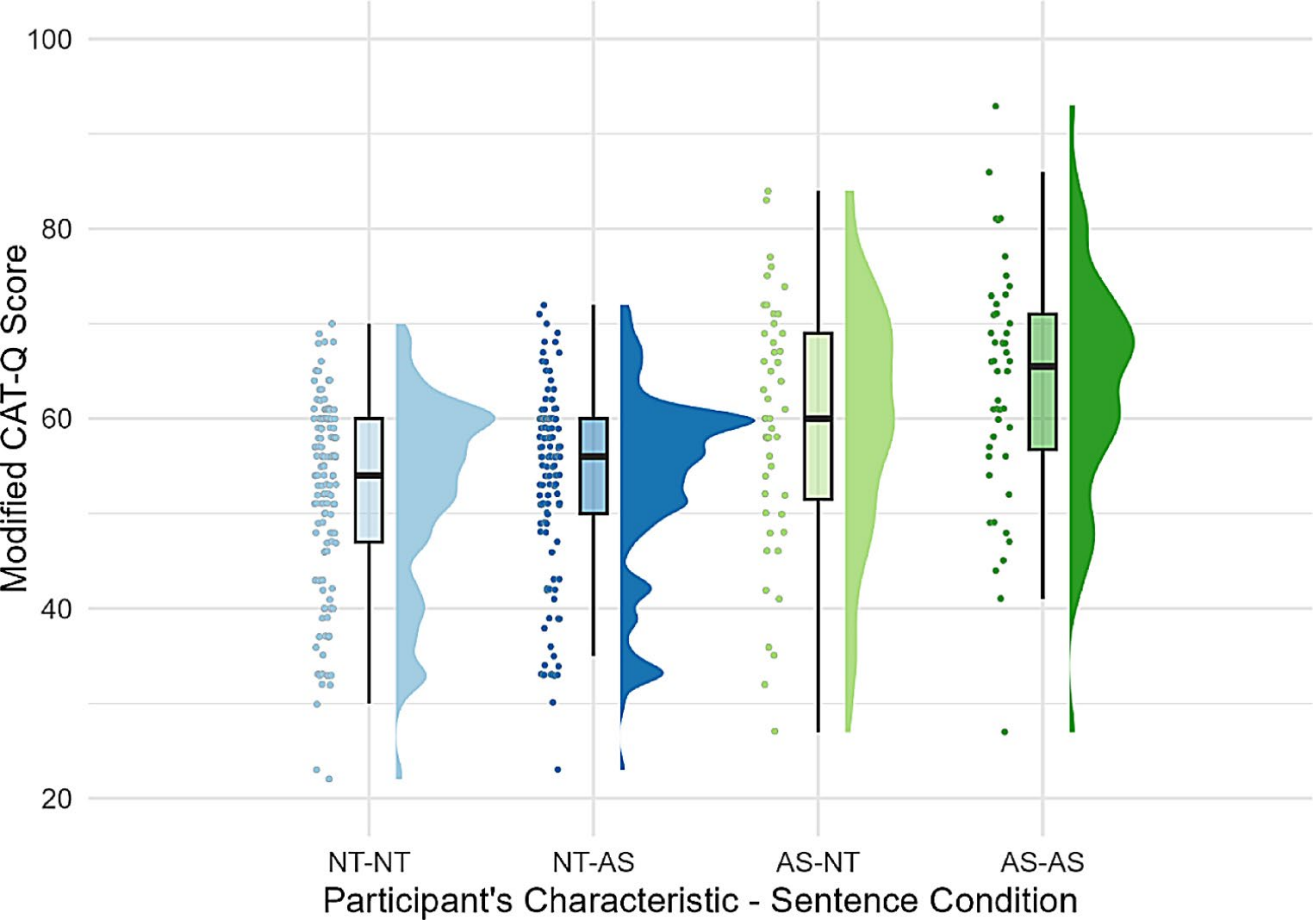
Distribution of modified CAT-Q Score by the Combination of Participants’ Characteristics and Sentence Condition. *Note. CAT-Q* = Camouflaging autistic traits questionnaire; A–*Β* indicates the modified CAT-Q score of group A in the *Β* sentence condition; NT-AS indicates the modified CAT-Q score of the NT group (participants) in the AS sentence condition (hypothetical interaction partner)

In both models, the main effect of the participants’ characteristics was significant (Model 1: *β* = 0.64, *p* < .001; Model 2: *β* = 0.69, *p* < .001), suggesting that a diagnosis of autism is associated with camouflaging. The interaction effect between participants’ characteristics and the sentence condition was also significant in both models (Model 1: *β* = 0.25, *p* = .037, Model 2: *β* = 0.25, *p* = .038), with subsequent simple slope analysis revealing that autistic participants were more likely to camouflage in the autistic sentence condition (Model 1: *β* = 0.36, *p* < .001; Model 2: *β* = 0.36, *p* < .001; Fig. 2). In contrast, the slope effect (the effect of the characteristics of an interaction partner) was not significant for neurotypical participants (Model 1: *β* = 0.11, *p* = .058; Model 2: *β* = 0.11, *p* = .058; Fig. 2).

In summary, the main effect of participants’ characteristics and the interaction effect between participants’ characteristics and sentence condition were significant in both models. The AS group exhibited greater camouflaging in the autistic sentence condition, whereas the NT group did not significantly differ in camouflaging levels in either condition.

### Ordinal Logistic Regressions of the Modified CAT-Q Individual Items

Ordinal logistic regression of individual modified CAT-Q items revealed that the AS group scored significantly higher than the NT group on 23 of the 30 items (Appendix 2). This is consistent with the results of the multilevel regressions, suggesting that the AS group engaged in camouflaging more than the NT group (Table 2). A closer qualitative examination of these significant items revealed substantial diversity in the descriptions of hypothetical interaction partners (derived from the SRS-2 for autistic and neurotypical interaction partners) and camouflaging behaviors (derived from the CAT-Q). This diversity suggests that the observed significantly higher modified CAT-Q scores in the AS group, compared with the NT group, were not exclusively influenced by a few specific or similar items, or in other words, neurodevelopmental characteristics or camouflaging behaviors. These findings suggest that the main effect of the multilevel regression is broadly supported by most items rather than a few items with strong effects. This was also the case when the items for the autistic and neurotypical sentence conditions were separated, with the AS group showing significantly higher camouflaging in 13 of 15 items in the autistic sentence condition and 10 of 15 items in the neurotypical sentence condition. This result suggests that the main and interaction effects were not exceedingly biased by individual items or the specific combination of original SRS-2 and CAT-Q items used to create the new set of items for this study.

In summary, ordinal logistic regression of individual modified CAT-Q items revealed that the AS group was more likely to camouflage across wide social contexts and with various behaviors.

## Discussion

In this study, we examined, for the first time, the influence of the autistic and neurotypical characteristics of interaction partners on camouflaging. Specifically, we examined whether autistic people camouflage more toward neurotypical others than toward autistic others. To test this hypothesis, we created a modified CAT-Q by combining it with SRS-2 items to measure camouflaging toward imagined autistic and neurotypical others separately. These items were administered to autistic and neurotypical participants and analyzed using multilevel regression. The analysis revealed that autistic individuals had significantly higher camouflaging scores than neurotypical individuals, consistent with previous studies (Dell’Osso et al., 2021, 2022; Hongo et al., 2022; Hull et al., 2019, 2020). Most critically, autistic individuals engaged in more camouflaging toward autistic others, which contrasts with neurotypical individuals who did not show a significant difference based on the characteristics of interaction partners.

This finding contradicts our initial hypothesis that autistic people would camouflage toward neurotypical others. One possible interpretation of this novel and counterintuitive finding is that autistic people are more motivated to interact with autistic others, leading to increased camouflaging to develop and maintain good relationships with autistic others, as the desire to interact with others is a primary motivation for camouflaging (Hull et al., 2017). Previous studies have indicated that autistic people prefer interacting with autistic others. Bolis et al. (2021) demonstrated that friends with similar levels of autistic traits had a higher perceived friendship quality, suggesting a preference for interaction among individuals with higher autistic traits even without verbal confirmation of their social counterparts being autistic. Chen et al. (2021) investigated autistic peer preferences in real-life settings with adolescents, revealing that autistic adolescents were more likely to interact with autistic peers than with neurotypical peers. This finding suggests that autistic peer preference begins at a relatively early age and in natural settings.

Based on this interpretation, camouflaging, previously conceptualized as a specific effort to bridge the “disjuncture” between autistic and neurotypical individuals by displaying more neurotypical behaviors, may be better understood as a broader social behavior aimed at projecting a favorable image to others. This view is consistent with discussions suggesting that camouflaging shares basic characteristics with social behaviors commonly observed in neurotypical individuals, such as impression management, albeit with autism-specific motivations and neurocognitive underpinnings (Ai et al., 2022). This implication raises the question of what distinguishes the camouflaging behavior of autistic individuals from the impression management employed by neurotypical individuals. Future research could address this question by adopting a comparative approach to examine the quality of social strategies employed by neurotypical and autistic populations, building on current studies that predominantly focus on the quantitative comparison of social strategies used by the respective groups.

Ordinal logistic regressions of individual items were conducted to ensure that the aforementioned findings were not driven by a few items within the modified CAT-Q (Appendix 4). The results revealed that, in 23 out of 30 items, the AS group exhibited significantly more camouflaging, supporting the main effect in multilevel regressions. When separating sentence conditions, the AS group showed more camouflaging in 13 of 15 items in the autistic sentence condition and 10 of 15 items in the neurotypical sentence condition. This finding suggests that the interaction effect observed in multilevel regressions did not depend on a few items with a strong effect but rather supported equally by most items, further strengthening the validity of our findings.

Although these findings are compelling, at least three limitations of our study should be acknowledged. Firstly, the autistic participants in this study may not be fully representative of the entire autistic population. Some of the participants in this study were recruited from peer groups and referrals from autistic individuals, in addition to support and service organizations; hence, some of them may have been more experienced and motivated to interact with autistic others. Secondly, our study was not formally pre-registered, even though we made every effort to minimize publication bias and ensure reproducibility within our limited resource. Future pre-registered replication studies would help test the reproducibility and robustness of the current findings. Finally, our study participants were primarily from a single cultural group (Japanese), and the sample size was relatively small to completely evaluate the effect of individual characteristics. Further studies should include participants from more diverse cultural backgrounds and evaluate the influence of cultural differences and individual characteristics on camouflage behavior.

In conclusion, this study examined how autistic and neurotypical individuals differ in camouflaging when interacting with others with autistic and neurotypical characteristics. Contrary to our initial hypothesis, autistic individuals engaged in more camouflaging toward others with autistic characteristics, while neurotypical individuals did not significantly differ in camouflaging toward others with autistic or neurotypical characteristics. This unexpected tendency of autistic people to camouflage more toward others with autistic characteristics could be explained by the higher social motivation of autistic people to interact with autistic others. Camouflaging might be better understood as a broader social behavior to improve their relationships with others (albeit with autism-specific motivations and neurocognitive underpinnings), rather than merely appearing “neurotypical” to survive in neurotypical-majority society. This study highlights the dynamic nature of camouflaging, regulated by a combination of autistic and neurotypical characteristics of interaction partners.

## APPENDIX

### Modified Camouflaging Autistic Traits Questionnaire (CAT-Q)

The following are the social situations and experiences in the given situations: If you were in such a social situation, would you have had the experience described below? Choose the answer that best describes your experience in the given social interaction.

1. Strongly Disagree.
2. Disagree.
3. Somewhat Disagree.
4. Neither Agree nor Disagree.
5. Somewhat Agree.
6. Agree.
7. Strongly Agree.

#### Practice Items

1. **There are people who take things too literally**,** misinterpreting the intended meaning of parts of a conversation. When interacting with these people**,** I feel like I’m “performing” rather than being myself.**
2. There are people who do well with adults outside of their families. When interacting with these people, I deliberately copy their body language or facial expressions.

#### Main Items

1. There are people who can express their thoughts smoothly and are calm during conversations. When interacting with these people, I adjust my body language or facial expressions so that I appear relaxed.
2. **There are people who engage in rigid or inflexible patterns of behavior that seem odd to people when under stress. When interacting with these people**,** I monitor my body language or facial expressions so that I appear interested by the person I am interacting with.**
3. **There are people who are thought to be emotionally distant and do not show their feelings to other people. When interacting with these people**,** I use behaviors that I have learned from watching other people interacting.**
4. **There are people who avoid those who want to be emotionally close to them. When interacting with these people**,** I need support of other people in order to socialize.**
5. **There are people who cannot respond appropriately to mood changes in others (for example**,** when a friend’s mood changes from happy to sad). When interacting with these people**,** I feel like I’m “performing” rather than being myself.**
6. **There are people whose way of greeting another person is unusual. If I were to interact with these people**,** I would practice my facial expressions and body language to make sure they look natural.**
7. **There are people who sometimes make the mistake of walking between two people who are trying to talk to one another. When interacting with these people**,** I have to force myself to interact with them.**
8. There are people who have good personal hygiene. When interacting with these people, I deliberately copy their body language or facial expressions.
9. There are people who are not regarded by others as odd or weird. When interacting with these people, I feel like the conversation flows naturally*.
10. There are people who deliberately join group activities or social events without being prompted or strongly urged to do so. When interacting with these people, I monitor my body language or facial expressions so that I appear relaxed.
11. There are people who have a good sense of humor and can understand jokes. When interacting with these people, I need the support of other people in order to socialize.
12. There are people whose facial expressions do not send the wrong message to others about how they actually feel. When interacting with these people, I try to find ways to avoid interacting with others.
13. **There are people who are not well coordinated. When interacting with these people**,** I feel free to be myself*.**
14. **There are people who do not recognize when something is unfair. When interacting with these people**,** I don’t feel the need to make eye contact if I don’t want to*.**
15. There are people who get well with family members. When interacting with these people, I always think about the impression I make on them.
16. There are people who realize by themselves when they get overly loud. When interacting with these people, I adjust my body language or facial expressions so that I appear relaxed.
17. **There are people who do not offer comfort to others when they are sad. When interacting with these people**,** I rarely feel the need to put on an act in order to get through*.**
18. **There are people who do not feel self-confident when interacting with others. When interacting with these people**,** I monitor my body language or facial expression so that I appear interested by them.**
19. **There are people who are not usually aware of how others are feeling. When interacting with these people**,** I will repeat phrases that I have heard others say in the exact same way that I first heard them.**
20. **There are people who are more suspicious than most people. If I were to interact with these people**,** I would spend time learning social skills from television shows and films**,** and try to use these in my interactions.**
21. There are people who do not mind with changes in their routine. When interacting with these people, I do not pay attention to what my face or body are doing*.
22. There are people who have good self-confidence. If I were to interact with these people, I would develop a script to follow in social situations (e.g., a list of questions or topics of conversation).
23. There are people who would rather be with others then being alone. I adjust my body language or facial expression so that I appear interested by the person I am interacting with.
24. **There are people who avoid starting social interactions with others. When interacting with these people**,** I feel like I am pretending to be “normal.”**
25. **There are people who are not interested in what others around them are paying attention to. When interacting with these people, I am always aware of the impression I make on them.**
26. There are people who concentrate on seeing the whole picture rather than on parts of things. If I were to interact with these people, I would research the rules of social interactions (for example, by studying psychology or reading books on human behavior) to improve my own social skills.
27. There are people who can answer questions directly and briefly end the conversation on the subject. When interacting with these people, I rarely feel the need to put on an act in order to get through a social situation*.
28. There are people who are not overly sensitive to certain sounds, textures, or smells. If I were to interact with these people, I would research the rules of social interactions (for example, by studying psychology or reading books on human behavior) to improve my own social skills.
29. **There are people who have more trouble than most people with understanding chains of causation (in other words**,** how events are related to one another). If I were to interact with these people**,** I would learn how people use their bodies and faces to interact by watching television or films**,** or by reading fiction.**
30. There are people who do not think or talk about the same thing over and over. When interacting with these people, I try to improve my understanding of social skills by watching other people.

*Note.* Items with asterisks (*) represent reverse items. Bolded items represent autistic sentence conditions, and non-bolded items represent neurotypical sentence conditions.

## Appendix 2

### Ordinal Logistic Regressions of Individual Modified CAT-Q Items

**Table:**

Item	Estimate	SE	*p*	q^a^
1	0.38	0.31	0.217	0.242
2	1.22**	0.33	< 0.001	0.001
3	1.81***	0.34	< 0.001	< 0.001
4	2.27***	0.36	< 0.001	< 0.001
5	1.22**	0.32	< 0.001	0.001
6	1.11**	0.33	0.001	0.002
7	0.96**	0.33	0.003	0.006
8	0.76*	0.31	0.016	0.023
9	-0.20	0.30	0.501	0.518
10	0.28	0.32	0.376	0.403
11	1.30***	0.33	< 0.001	< 0.001
12	1.15**	0.33	0.001	0.002
13	-1.00**	0.32	0.002	0.004
14	-0.87*	0.32	0.007	0.011
15	0.76*	0.32	0.016	0.023
16	0.70*	0.32	0.031	0.040
17	-0.66	0.32	0.042	0.053
18	1.37***	0.32	< 0.001	< 0.001
19	1.54***	0.34	< 0.001	< 0.001
20	1.18***	0.33	< 0.001	0.001
21	-0.20	0.31	0.518	0.518
22	1.06**	0.32	0.001	0.003
23	1.04**	0.33	0.002	0.004
24	1.05**	0.34	0.002	0.005
25	0.96**	0.33	0.004	0.007
26	1.51***	0.34	< 0.001	< 0.001
27	-0.81*	0.32	0.013	0.020
28	0.79**	0.33	0.017	0.023
29	0.57	0.32	0.077	0.092
30	0.44	0.32	0.158	0.182

*Note.* *N* = 185 (AS group, *n* = 48; NT group, *n* = 137). Positive estimate values indicated greater camouflaging in the AS group

^a^ corrected p-value with the false-discovery method

**p* < .05, ***p* < .01, ****p* < .001,

Autistic sentence condition = 2, 3, 4, 5, 6, 7, 13, 14, 17, 18, 19, 20, 24, 25, and 29

Neurotypical sentence condition = 1, 8, 9, 10, 11, 12, 15, 16, 21, 22, 23, 26, 27, 28, and 30
